# The molecular epidemiology of incident methicillin-resistant *Staphylococcus aureus* cases among hospitalized patients in Alberta, Canada: a retrospective cohort study

**DOI:** 10.1186/s13756-015-0076-1

**Published:** 2015-09-14

**Authors:** Kathryn Bush, Jenine Leal, Sumana Fathima, Vincent Li, David Vickers, Linda Chui, Marie Louie, Geoffrey Taylor, Elizabeth Henderson

**Affiliations:** Infection Prevention and Control, Alberta Health Services, Calgary, AB Canada; Alberta Provincial Laboratory for Public Health, Edmonton and Calgary, AB Canada; Department of Laboratory Medicine and Pathology, University of Alberta, Edmonton, AB Canada; Department of Microbiology Immunology and Infectious Diseases, University of Calgary, Calgary, AB Canada; Department of Medicine, University of Alberta, Edmonton, AB Canada; Department of Community Health Sciences, University of Calgary, Calgary, AB Canada

**Keywords:** MRSA epidemiology, Infection prevention and control, Community-associated MRSA, Healthcare-associated MRSA

## Abstract

**Background:**

Infection Prevention and Control (IPC) surveillance for incident methicillin-resistant *Staphylococcus aureus* (MRSA) in hospitalized patients is performed in a complete provincial surveillance network of all acute care facilities in Alberta, Canada. IPC surveillance is centralized using a web-based data entry platform so that each patient is counted only once. All diagnostic laboratories submit the first clinical MRSA isolate associated with a patient without previous MRSA positive clinical cultures in the preceding year to the Provincial Laboratory for Public Health (ProvLab) for molecular typing. This study will investigate the relationship between the IPC epidemiological classification based on time of detection following admission to hospital (Hospital Acquired and Community Associated) and the matched laboratory MRSA surveillance data using a retrospective cohort study design.

**Methods:**

Incident IPC MRSA cases were classified according to IPC epidemiologic definitions. DNA sequencing of the *Staphylococcus* protein A (*spa*) gene and pulsed-field gel electrophoresis (PFGE) typing was performed. IPC MRSA surveillance data were matched to the ProvLab molecular surveillance data. Univariate comparisons of proportions were performed for categorical variables and the Student’s *t* test for continuous variables.

**Results:**

MRSA molecular typing data were available for matching for 46.7 % (2248/4818) of incident IPC cases. There was agreement in definitions for traditional nosocomial clones (USA100/CMRSA2) with Hospital Acquired (HA)-MRSA (65.1 % of all IPC HA-MRSA) and traditional community clones (USA400/CMRSA7 and USA300/CMRSA10) with Community Acquired (CA)-MRSA (62.4 % of CA-MRSA). However, we observed discordance for both traditional nosocomial/CA-MRSA (30.4 % of CA-MRSA) and for traditional community/HA-MRSA (26.9 % of HA-MRSA).

**Conclusions:**

We note agreement between traditional nosocomial clones and HA-MRSA, and traditional community clones and CA-MRSA. However, approximately one-quarter of HA-MRSA are those of traditional community clones while approximately one-third of CA-MRSA are those of traditional nosocomial clones. Collaborative provincial MRSA surveillance is important as the distinction between IPC case attribution in acute care settings and the historical definitions of MRSA clones as community- or healthcare-associated have blurred.

## Background

Methicillin-resistant *Staphylococcus aureus* (MRSA) is a major cause of healthcare-associated infections. In Canada there are ten recognized epidemic types of MRSA based on pulsed-field gel electrophoresis (PFGE) patterns (CMRSA1–10) [1]. In Canadian healthcare facilities, MRSA strains associated with the USA100/CMRSA2 clone have predominated [2]. However, traditionally community-associated USA400/CMRSA7 and USA300/CMRSA10 clones are becoming more common in the healthcare setting [3]. Reports on the epidemiology of MRSA have described the blurring in the definitions of traditional nosocomial- and traditional community-MRSA clones [3–6].

Surveillance for healthcare-associated infections is a mandate for Infection Prevention and Control (IPC) programs to establish baseline frequency of disease, identify risk factors, measure the impact of prevention initiatives, and provide information to inform and educate healthcare workers [6]. Surveillance is most successful when it is comprehensive and linked to program objectives so that surveillance reports are timely and subsequent actions are meaningful and addressed [7].

The IPC program in Alberta, Canada conducts surveillance of healthcare-associated infections in a complete network with participation from every acute care facility in the province. The IPC surveillance program uses epidemiological definitions based on time to detection of the MRSA from the admission date of the patient to the facility. These definitions categorize the incident MRSA as community or healthcare-related. An incident IPC case can include a colonized or an infected patient.

A core function of the Provincial Laboratory for Public Health (ProvLab) is laboratory surveillance, which includes the identification of emerging problems, reporting strategies, dissemination of information and other intervention programs for infectious diseases. MRSA is a notifiable pathogen in Alberta and molecular typing of first clinical MRSA isolate in a patient in the preceding 12 months has been performed at the ProvLab since June 2005 [4]. Historically, MRSA typing in Alberta has used pulsed-field gel electrophoresis (PFGE) types however in March 2010 *Staphylococcus aureus* protein A (*spa*) typing was implemented, with standardized nomenclature that allows for easy international comparisons [8].

In this paper we discuss the molecular epidemiology of MRSA cases detected by an extensive, collaborative surveillance network in the Canadian province of Alberta.

## Methods

### Study population

The province of Alberta, Canada had a population of 3,785,597 in 2011 in a total geographic area of 661,190 square kilometers. Most (68.5 %) of the population reside in geographic areas surrounding two major urban centers: Calgary (2011 population 1,408,606; 37.2 % of the provincial total) and Edmonton (1,186,121; 31.3 %). Created in 2009, Alberta Health Services (AHS) provides the majority of healthcare in the province along with a partner provider agency, Covenant Health (COV). AHS/COV provided 2,985,493 hospital patient-days across all hospital facilities in 2011 (40.4 % in the Edmonton zone, 28.7 % in the Calgary zone).

### Ethics statement

IPC conducts mandatory surveillance to monitor healthcare-associated infections in all acute care facilities in the province. A Project Ethics Community Consensus Initiative was completed. Based upon six ethical considerations, the project was deemed to be under the IPC mandate for quality improvement and approved to meet ethical standards; written consent was not required for this analysis. The project data were collected in the provincial IPC surveillance database in patient identified form, however, for analysis all data were deidentified and project results are presented in aggregate format. Existing privacy impact agreements between the ProvLab and Alberta Health Services enabled analysis of patient-level data.

### Surveillance network

Infection Control Professionals in AHS/COV began monitoring MRSA in admitted patients at all acute care facilities in Alberta on April 1, 2011 by reviewing all positive laboratory cultures from hospitals throughout the province. Provincial IPC surveillance is based on national surveillance protocols. An incident MRSA case is defined as the first time a patient has a confirmed positive MRSA culture from a screening or clinical specimen while admitted to an AHS/COV acute care facility. IPC surveillance is centralized using a web-based data entry platform and patient transfers in all acute care facilities are monitored so that each patient is counted only once. Infection Control Professionals review patient charts to collect epidemiologic data to classify the MRSA. Patients in acute care hospitals in Alberta are screened for MRSA according to provincial guidelines [9].

All acute care laboratories submit the first clinical MRSA isolate associated with a patient without previous MRSA positive clinical cultures in the preceding year to ProvLab for molecular typing.

### IPC MRSA classification

An acute care patient who is newly identified with MRSA is an incident IPC case and is classified based on the following criteria as has been described [10]: HA-MRSA identified more than 48 h after admission; HCA within 48 h after admission AND healthcare risk factors (such as Long Term Care resident or renal patient); CA-MRSA within 48 h after admission and does not fulfill criteria for HA or HCA.

Additional data elements were collected as part of routine IPC surveillance. Demographic information included the patient’s date of birth and gender; and other data elements included admission date, ordering acute care facility, specimen site, and whether the specimen was taken as a screening specimen or as part of clinical work-up. Decisions on case severity (infection or colonization) are according to definitions from the National Healthcare Safety Network (NHSN) [11].

### Strain typing

ProvLab performs PFGE as described by Mulvey et al. [1]; PFGE patterns generated were entered into the MRSA database and analysed using the BioNumerics software (version 5.1; Applied Maths, Texas, US). PFGE profiles were grouped into CMRSA epidemic clones according to the Canadian MRSA classification system [8]. The terms “traditionally nosocomial” and “traditionally community” MRSA strains were adapted from Hudson [5] for this study to denote clones that were originally identified as healthcare reservoir (i.e., USA100/CMRSA2) or community-associated (i.e., USA400/CMRSA7 and USA300/CMRSA10).

Polymerase Chain Reaction (PCR) for *spa* typing was performed using primers targeting the *spa* gene as previously described [8, 12, 13]. PCR products were purified using the ChargeSwitch PCR Clean-up Kit (Invitrogen, Canada) and sequenced using the BigDye Terminator v3.1 Cycle Sequencing Kit (Life Technologies, Canada). *spa* typing sequencing results were analyzed using BioNumerics and submitted to the online Ridom *spa* database (http://spaserver.ridom.de/), developed by Ridom GmbH and curated by SeqNet.org (http://www.SeqNet.org), for Ridom *spa* type designation and assignment of Ridom repeat successions [12].

### Data matching and analysis

**Fig. 1:**
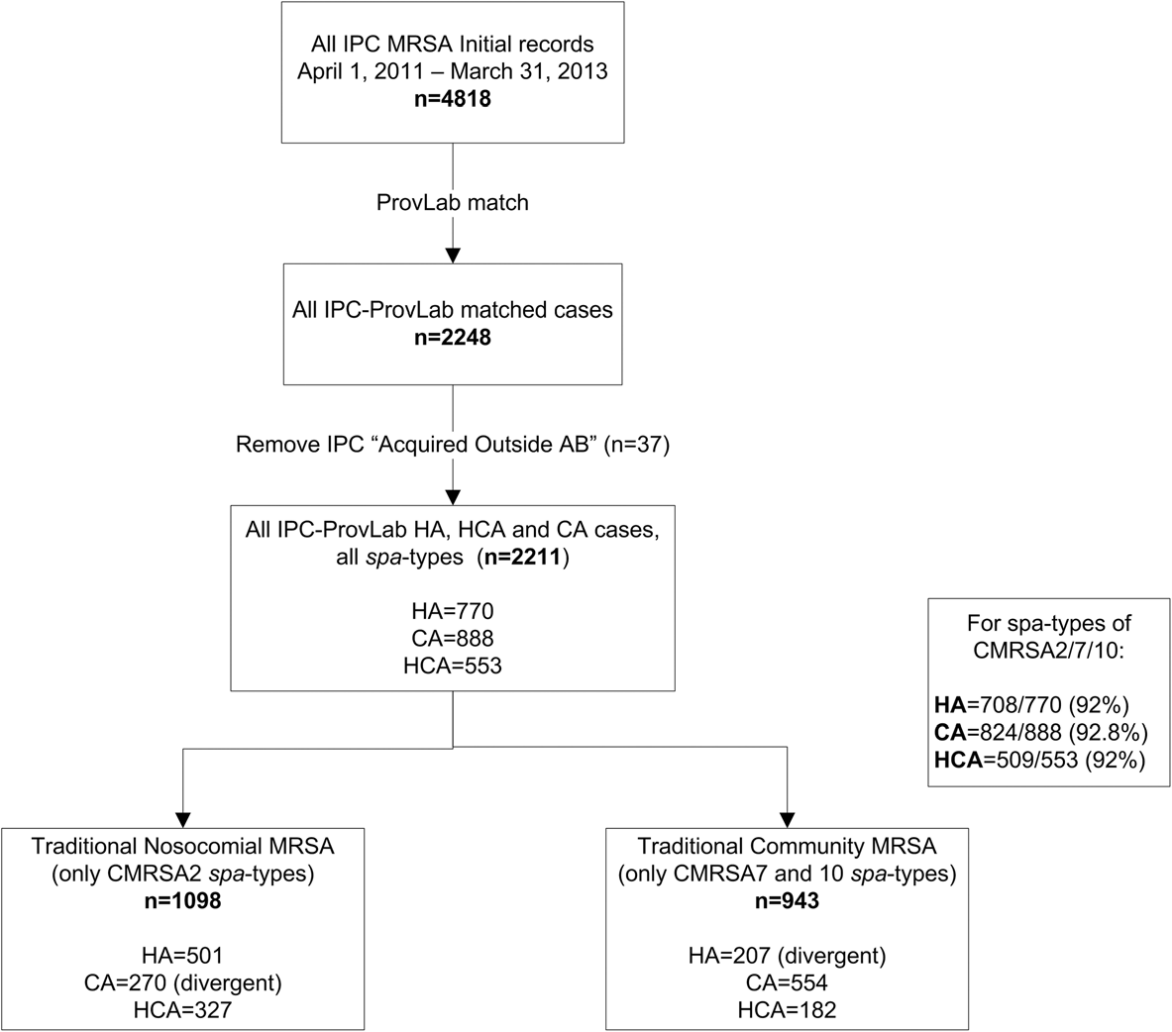
Data analysis flowchart

The IPC data for the 2 year study period between April 2011-March 2013 were extracted and included in the analysis. The IPC data included incident MRSA cases among hospitalized patients. Since ProvLab types only one MRSA clinical isolate per patient in a year from any community or acute care setting, additional ProvLab data (i.e., from January 2010-March 2013) were included. The IPC and the ProvLab data were linked by deterministic matching in a one-to-many relationship using each patient’s provincial healthcare number, last name, first name, and date of birth. The ProvLab clinical isolate nearest in date to the incident IPC case identified from a hospital-collected clinical or screening specimen was selected for analysis (Fig. 1). An incident IPC screening case identified from a screening specimen was included if the patient had any clinical specimen that was sent for molecular testing in the preceding year. Thus, all IPC-ProvLab matched data represent MRSA typing results from a clinical specimen.

**Fig. 2:**
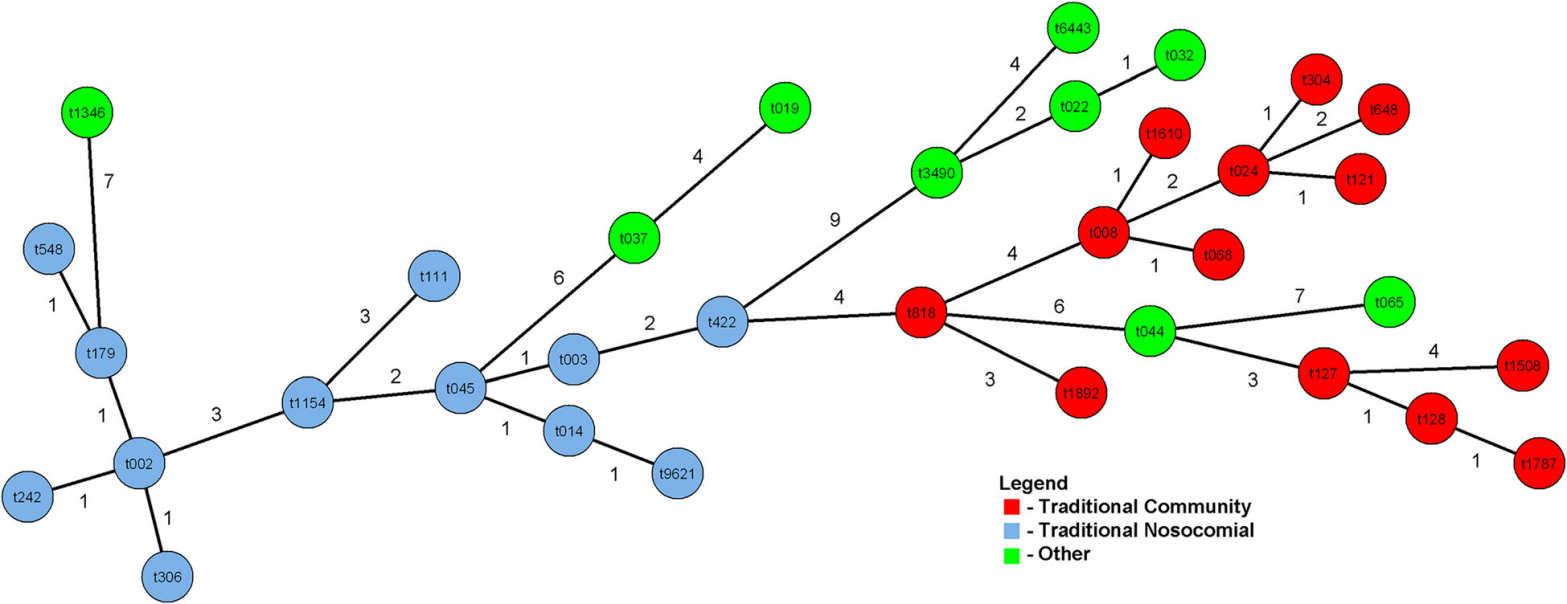
Minimum Spanning Tree of MRSA *spa* types observed at least 5 times

A Minimum Spanning Tree (MST) of MRSA *spa* types observed at least five times in Alberta for the IPC-matched data was constructed using BioNumerics (Fig. 2). Cluster analysis was performed using the duplications, substitutions, indels (DSI) algorithm in BioNumerics with the following parameters: 250 % gap creation cost; 50 % gap extension, duplicate creation and duplicate extension costs; maximum three repeat duplication; and 1.00 % bin grouping distance. Each circle in Fig. 2 represents a different *spa* type and similarity values between *spa* types are shown adjacent to the connecting lines. IPC case classifications associated with each *spa* type are shaded as indicated in the figure legend.

Data were analyzed using STATA/IC 10.0 (StataCorp, Texas, 2007). Univariate comparisons of proportions were performed for categorical variables and the Student’s *t* test for continuous variables. MRSA rates were calculated by dividing the number of incident HA-MRSA cases by the total hospital patient-days per year per 10,000; and CA and HCA-MRSA cases by the hospital admissions per year per 1000 as per IPC rate reporting conventions [14]. For all statistical comparisons a *p*-value < 0.05 was deemed statistically significant.

## Results

Rates were stable across the study period: the provincial HA-MRSA rates were 3.1 per 10,000 patient-days in 2011–12 and 2.8 per 10,000 patient-days in 2012–13; the provincial HCA-MRSA rates were 1.8 per 1000 admissions in both years; and the provincial CA-MRSA rates were 2.5 per 1000 admissions in both years. MRSA cases are detected primarily through patient screening: 67.4 % (3249/4818) of all isolates were detected by screening swabs (i.e., nares/groin/axilla). See Table 1 for case counts of HA, HCA and CA-MRSA.

**Table 1:** Comparison of all IPC MRSA cases to IPC-ProvLab matched data

April 2011 – March 2013 n(%)	All IPC MRSA data		IPC-ProvLab matched data		*p* value*
	*n* = 4818		*n* = 2211		
	Clinical *n* = 1569 (32.7)	Screening *n* = 3249 (67.4)	Clinical *n* = 1393 (63.0)	Screening *n* = 818 (37.0)	< 0.05
Gender					0.25
Male	825 (52.6)	1699 (52.3)	724 (52.0)	446 (54.5)	
Age (Years)					< 0.05
Mean Age (±SD)	57 (25.4)	66 (22.8)	56 (25.1)	63 (22.6)	
Median Age (IQR)	61 (18.1)	73 (11.4)	59 (18.2)	67 (14.9)	
Case Classification					
Hospital Acquired	645 (41.1)	1077 (33.1)	578 (41.5)	192 (23.5)	< 0.01**
Healthcare	326 (20.8)	978 (30.1)	277 (19.9)	276 (33.7)	< 0.04**
Associated					
Community Acquired	598 (38.1)	1194 (36.7)	538 (38.6)	350 (42.8)	< 0.01**
Facility Bed Size					
>500	681 (43.4)	1847 (56.8)	621 (44.6)	460 (56.2)	0.75
251–500	285 (18.2)	495 (15.2)	245 (17.6)	144 (17.6)	0.1
100–250	233 (14.9)	334 (10.3)	210 (15.1)	87 (10.6)	0.76
<100	370 (23.6)	573 (17.6)	317 (22.8)	127 (15.5)	0.15
Severity					
Infected	1432 (91.3)	n/a	1274 (91.5)	n/a	0.86
Clinical Culture Site					
Blood	142 (9.1)	n/a	130 (9.3)	n/a	0.79
Other Sterile	89 (5.7)		88 (6.3)		0.45
Skin/Soft Tissue	947 (60.4)		830 (59.6)		0.67
Infected Device	35 (2.2)		29 (2.1)		0.78
Respiratory	193 (12.3)		172 (12.3)		0.97
Urine	163 (10.4)		144 (10.3)		0.96

All incident IPC MRSA cases can be identified from clinical and screening isolates. Matched clinical isolates from the ProvLab data repository were used as the surrogate molecular type for IPC cases identified from “screening” cultures

*Tests of significance compare the “screening” populations, except for Clinical Culture site data

**Bonferroni correction adjusted level of significance (*α* = 0.017) used to consider multiple comparisons with mutually exclusive groups

ProvLab typing data were available for 46.7 % (2248/4818) of incident IPC cases. Those MRSA cases acquired outside Alberta (*n* = 37) were not included in this analysis. Of the IPC-ProvLab matched cases (*n* = 2211), 34.8 % were classified as HA, 25.0 % as HCA and 40.2 % as CA-MRSA.

The IPC data were compared to the sub-set of IPC-ProvLab matched data to see how well the matched data represented the IPC surveillance data (Table 1). The data were stratified based on the origin of the incident IPC case (clinical or screening specimen), however all patients in the IPC-ProvLab matched data had MRSA typing results from a clinical specimen even when the incident IPC case was from a screening specimen. Eighty-eight per cent (1393/1569) of the IPC clinical specimens had a match in the ProvLab data. Clinical specimens in the matched and unmatched data sets therefore had no significant differences in gender, age, IPC classification or specimen type. Most of these specimens were deemed infections (91 % in both the matched and unmatched data sets) according to NHSN definitions. For the screening data, 25.2 % (818/3249) of the IPC data had a match in the ProvLab data. These patients were younger and more likely to be CA according to the IPC surveillance definitions.

Figure 2 displays the genetic relationships among *spa* types detected at least five times in the IPC-ProvLab matched data. Isolates belonging to the same epidemic clone clustered together and clear distinctions are observed between them. Four *spa* types comprise 73.2 % of all isolates: t002 (USA100/CMRSA2, 698/2211, 31.6 %), t008 (USA300/CMRSA10, 647/2211, 29.3 %), t003 (USA100/CMRSA2, 165/2211, 7.5 %) and t128 (USA400/CMRSA7, 107/2211, 4.8 %) were the most common in the matched data.

**Fig. 3:**
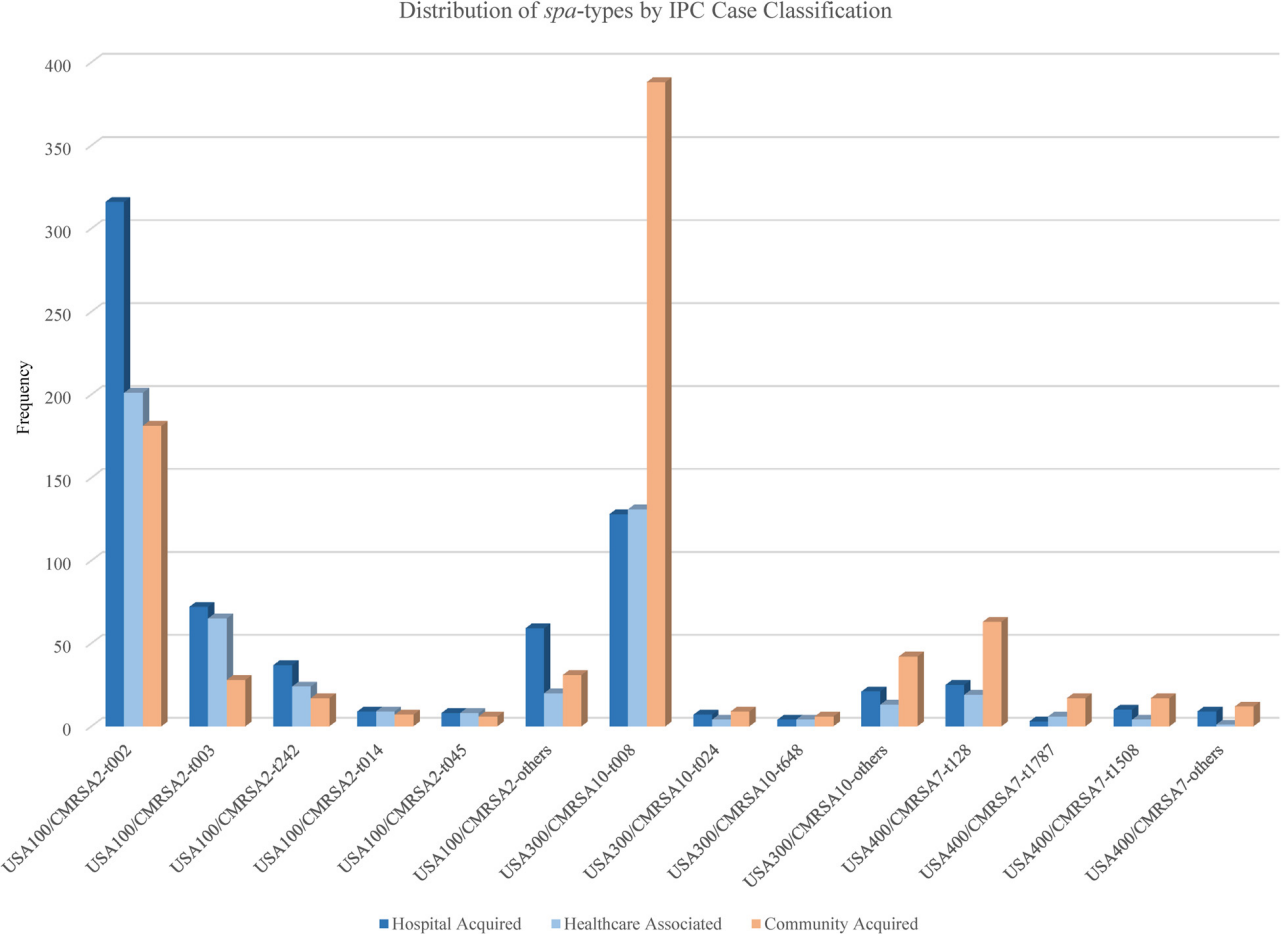
IPC MRSA case classification and *spa* type distribution

The relationship of specific *spa* types to PFGE nomenclature and IPC classification are displayed in Fig. 3 with additional data provided in the Appendix. The “Other *spa* types” category for each PFGE type are a heterogeneous mix,. For example, the CMRSA2 “Other *spa* types” comprise 10 % of this clone with more than 40 unique *spa* types present in this category. The same is true for USA300/CMRSA10, where 28 *spa* types are identified for 76 isolates. Sixty-five percent of HA-MRSA were primarily traditional nosocomial MRSA, with *spa* type t002 (316/501, 63.1 %) being most common. CA-MRSA were primarily traditional community MRSA (554/888, 62.4 %). The HCA category, denoting other healthcare risks, was a mix of traditional nosocomial and traditional community MRSA. There was discordance seen for IPC-defined HA-MRSA with traditional community MRSA (USA400/CMRSA7 and USA300/CMRSA10, 207/770, 26.9 %) and for IPC-defined CA-MRSA with traditional nosocomial (USA100/CMRSA2, 270/888, 30.4 %).

## Discussion

In this paper, we describe the molecular epidemiology of MRSA cases in acute care patients detected by an extensive, collaborative surveillance network in the Canadian province of Alberta. Surveillance for healthcare-associated infections such as MRSA is deemed an essential component of any IPC program and is included as a required organizational practice for Canadian healthcare systems [15]. The Public Health Agency of Canada notes that a region-wide database is needed to identify and track healthcare-associated infection trends as patients move between healthcare facilities and healthcare settings [16]. This type of surveillance however, requires effective Infection Control Professionals that cooperate and communicate among the various settings and facilities to ensure standardized data collection, analysis, and interpretation methods so that the reported rates and estimated costs of healthcare-associated infections are reliable. With the formation of a single health delivery entity in 2009, all provincial hospital IPC surveillance programs consolidated to a single provincial program. This surveillance system is centered on a web-based data entry platform that enables patient-level data to be available to Infection Control Professionals for patient management purposes. Standardized provincial surveillance definitions maintain data consistency, enable facilities to benchmark, and allows for comparisons of disease trends over time. These, in turn, are used to help create a safer and healthier environment for patients and their families, and healthcare providers.

The IPC-ProvLab matched specimens where the incident IPC case was from a clinical specimen represents the majority of clinical specimens in the IPC data. When IPC identified an incident MRSA case from a screening specimen, the IPC-ProvLab matched dataset was able to find a clinical MRSA isolate within the year of the IPC case presentation. The ProvLab testing protocol assumes stability in the patient’s MRSA clone, so that the MRSA from a screening specimen is assumed to be the same clone as the one typed from a clinical isolate within the preceding year. There is support for this testing assumption in the literature, with duration of MRSA colonization of more than 200 days described [17]. In a recent longitudinal evaluation of MRSA colonized patients with an average of five MRSA isolates over an average of 251 days, there was 85.7 % genetic concordance seen within patients’ individual isolates [18].

It is important to consider both the epidemiologic definition as well as molecular typing when classifying MRSA in the healthcare setting to understand MRSA transmission dynamics. Laboratory testing using *spa* typing is useful because of its high concordance with PFGE epidemic types and allows international comparisons to be made, rather than relying on naming differences between Canadian, European, US and other naming conventions [8].

In our data, acute care patients who have a clinical isolate detected as positive in the community rather than during their acute care admission are younger and more likely to be identified as CA or HCA in the IPC data, since they may have already been identified in their patient record as MRSA positive and therefore screened on admission to the facility. Traditional community MRSA strains were first observed as community infections in Canada in the mid-1990’s. MRSA isolated from sentinel Canadian acute care sites from 1995 to 2007 showed USA300/CMRSA10 as the second most commonly isolated clone (27 % of isolates), with the majority seen in Western Canada [3]. An Alberta study from 2005 to 2008 confirmed this increase in USA400/CMRSA7 and USA300/CMRSA10 clones: the highest rates of USA300/CMRSA10 were reported in people aged 25–44, while highest rates of USA100/CMRSA2 were seen in patients >65 years [4]. The distribution of the most commonly isolated *spa* types were t002, t008, and t128 and they belonged to PFGE epidemic types USA100/CMRSA2, USA300/CMRSA10, and USA400/CMRSA7 respectively [4]. Our data were similar with t002 (31.6 % of all *spa* types), t008 (29.3 %), t003 (7.5 %) and t128 (4.8 %) being the predominant *spa* types. In the MST (Fig. 2) we have separated the *spa* types observed in hospitalized patients into categories to show the clustering of both the traditional nosocomial *spa* types and of the traditional community *spa* types, demonstrating the phylogenetic diversity with the Alberta MRSA strains. Traditional community *spa* types further cluster into USA400/CMRSA7 and USA300/CMRSA10 epidemic types, and the evolutionary dynamics between closely related *spa* types can be inferred.

We used the terminology of “traditional nosocomial” and “traditional community” to explore the mixing of MRSA clone reservoirs in relationship to the case classifications used in IPC surveillance definitions (HA, HCA and CA-MRSA) [5]. In Alberta, these are assigned based on the timing of the incident MRSA specimen in respect to the patient’s admission date or suspected epidemiological links in the acute care facilities. We noted agreement in definitions for traditional nosocomial clones (USA100/CMRSA2) with HA-MRSA (65.1 % of all HA-MRSA) and traditional community clones (USA400/CMRSA7 and USA300/CMRSA10) with CA-MRSA (62.4 % of all CA-MRSA). However, we observed discordance for both traditional nosocomial/CA-MRSA (30.4 % of CA-MRSA strains) and for traditional community/HA-MRSA (26.9 % of HA-MRSA strains). These findings are the focus of further study to investigate patients with traditional nosocomial/CA-MRSA; this may highlight areas for revisions of both the provincial IPC and national case classifications and surveillance definitions. For patients with traditional community/HA-MRSA, further work may highlight issues with admission screening guidelines in acute care facilities across the province and phylodynamic studies may identify clusters of endemic traditional community MRSA strains in our acute care facilities, as has been reported in other settings [6]. Understanding these populations better will inform standardized MRSA screening guidelines for all acute care admissions in the province as well as providing insights into the most effective prevention strategies in the healthcare setting.

### Study strengths

This study is unique since all acute care facilities are represented: including tertiary, smaller urban facilities as well as small remote rural locations. Because of the integrated nature of the IPC surveillance network, patient transfers are monitored across all facilities and a patient is only counted once in the surveillance network. All clinical isolates in the province are submitted to the ProvLab for molecular testing since June 2005. ProvLab holds a comprehensive laboratory based MRSA data repository, with data collated from submitting acute care laboratories. This is a unique opportunity to look at two separate standardized surveillance systems exploring complementary aspects for enhanced and integrated MRSA surveillance.

### Study limitations

Admission screening for MRSA is based on provincial guidelines [9], however facilities may devise screening strategies based on local MRSA epidemiology; so, all patients may not have an equal chance of being detected on admission to an acute care facility. Patients may be identified as HA-MRSA during their admission or may remain undetected and misclassified as CA-MRSA at some time more than 12 months after their first healthcare encounter. In addition, the proportion of colonization is influenced by variation in patient screening practices which may account for some differences in HA-MRSA rates between the facilities. Work is underway in the province to standardize MRSA screening and monitor compliance with those guidelines, however the exact compliance with current guidelines is not known.

While decision on infection was made using NHSN definitions [11], the majority of clinical specimens were determined to be infected. On-going data quality assessment and education on the use of the definitions is continuing; however there may have been over-call of infection in these data. We do not have information on how culturing practices for infections differ between sites, but our presumption is that they are similar since healthcare is provided in the same way across the province and many patient care best practices are standardized across all facilities.

Only the first clinical isolate per year is submitted by a regional laboratory for typing at ProvLab. Therefore a patient could acquire another MRSA clone within the year and this would not be identified. ProvLab does not type screening isolates so typing data were not available for incident IPC cases from screening specimens, and this study cannot rule out the possibility of a patient having colonization and infection with different MRSA strains.

## Conclusions

The molecular epidemiology of MRSA cases in acute care patients as detected by an extensive, collaborative surveillance network in the Canadian Province of Alberta. We note agreement between traditional nosocomial clones and HA-MRSA, and traditional community clones and CA-MRSA. However, approximately one-quarter of HA-MRSA are those of traditional community clones while approximately one-third of CA-MRSA are those of traditional nosocomial clones. Collaborative provincial MRSA surveillance is important as the distinction between IPC case attribution in acute care settings and the historical definitions of MRSA clones as community- or healthcare-associated have blurred and the original labels of “nosocomial” and “community” for molecular clones of MRSA are no longer correct in our setting. Further collaborations between IPC Surveillance-ProvLab teams will focus on the relationships between genetic types and IPC surveillance case classifications, in particular those cases where there is discordance between IPC case classification definitions and traditional settings of MRSA types. This work will highlight the need for effective IPC interventions in the community to reduce entrance of traditional community MRSA into hospital, as well as informing acute care interventions for patients around patient screening and additional isolation precautions.

## Appendix

IPC-ProvLab matched data showing *spa* type distribution of traditional nosocomial and traditional community MRSA clones by PFGE profile and by IPC case classification categories. This table supplements information provided on specific *spa* types in Fig. 3.

**Table 2:** MRSA case classification and molecular epidemiology

Matched MRSA April 2011 – March 2013 *n* = 2211		Hospital Acquired (HA) *n* = 770 (34.8 %)		Healthcare Associated (HCA) *n* = 553 (25.0 %)		Community Acquired (CA) *n* = 888 (40.2 %)	
		PFGE n (% of HA)	*spa* type n (% of PFGE)	PFGE n (% of HCA)	*spa* type n (% of PFGE)	PFGE n (% of CA)	*spa* type n (% of PFGE)
Traditional Nosocomial *n* = 1098	t002 (CMRSA 2)	501 (65.1)	316 (63.1)	327 (59.1)	201 (61.5)	270 (30.4)	181 (67.0)
	t003 (CMRSA 2)		72 (14.4)		65 (19.9)		28 (10.4)
	t242 (CMRSA 2)		37 (7.4)		24 (7.3)		17 (6.3)
	t014 (CMRSA 2)		9 (1.8)		9 (2.8)		7 (2.6)
	t045 (CMRSA 2)		8 (1.6)		8 (2.5)		6 (2.2)
	Other *spa* types (CMRSA 2)		59 (11.8) *n* = 25 *spa* types		20 (6.1) *n* = 14 *spa* types		31 (11.5) *n* = 22 *spa* types
Traditional Community *n* = 943	t008 (CMRSA 10)	160 (20.8)	128 (80.0)	152 (27.5)	131 (86.2)	445 (50.1)	388 (87.2)
	t024 (CMRSA 10)		7 (4.4)		4 (2.6)		9 (2.0)
	t648 (CMRSA 10)		4 (2.5)		4 (2.6)		6 (1.3)
	Other *spa* types (CMRSA 10)		21 (13.1) *n* = 8 *spa* types		13 (8.9) *n* = 13 *spa* types		42 (9.4) *n* = 24 *spa* types
	t128 (CMRSA7)	47 (6.1)	25 (53.2)	30 (5.4)	19 (63.3)	109 (12.3)	63 (57.8)
	t1787 (CMRSA7)		3 (6.4)		6 (20.0)		17 (15.6)
	t1508 (CMRSA7)		10 (21.3)		4 (13.3)		17 (15.6)
	Other *spa* types (CMRSA7)		9 (19.1) *n* = 8 *spa* types		1 (3.3) (*spa* type t175)		12 (11.0) *n* = 7 *spa* types
Others *n* = 170	t065 (CMRSA1)	2 (0.3)	2 (100.0)	9 (1.6)	4 (44.4)	3 (0.3)	2 (66.7)
	Other CMRSA1 *spa* types		0 (0.0)		5 (55.6) *n* = 3 *spa* types		1 (33.3) (*spa* type t026)
	t037 (CMRSA3/6)	11 (1.4)	11 (100.0)	2 (0.4)	2 (100.0)	6 (0.7)	6 (100.0)
	CMRSA4 *spa* types	2 (0.3)	2 (100.0) *n* = 2 *spa* types	0 (0.0)	0 (0.0)	0 (0.0)	0 (0.0)
	CMRSA5 *spa* types	1 (0.1)	1 (100.0) (*spa* type t064)	1 (0.2)	1 (100.0) (*spa* type t064)	1 (0.1)	1 (100.0) (*spa* type t1476)
	t022 (CMRSA8)	31 (4.0)	10 (32.2)	19 (3.4)	9 (47.4)	12 (1.4)	1 (8.3)
	t6443 (CMRSA8)		14 (45.2)		3 (15.8)		2 (16.6)
	Other CMRSA8 *spa* types		7 (22.6) *n* = 6 *spa* types		7 (36.8) *n* = 4 *spa* types		9 (75.0) *n* = 7 *spa* types
	t019 (PFGE Not Assigned)	15 (1.9)	1 (6.7)	13 (2.4)	0 (0.0)	42 (4.7)	17 (40.5)
	Other PFGE Not Assigned *spa* types		14 (93.3) *n* = 11 *spa* types		13 (100.0) *n* = 11 *spa* types		25 (59.5) *n* = 16 *spa* types

## Abbreviations

AHS/COV: Alberta Health Services/Covenant Health
CA: Community acquired
CMRSA: Canadian Methicillin Resistant *Staphylococcus aureus* classification system
HA: Hospital acquired
HCA: Healthcare associated
IPC: Infection prevention and control
MRSA: Methicillin resistant *Staphylococcus aureus*
MST: Minimum spanning tree
NHSN: National healthcare safety network
PCR: Polymerase chain reaction
PFGE: Pulsed-field gel electrophoresis
ProvLab: Alberta Provincial Laboratory for Public Health
*spa*: *Staphylococcus* protein A gene
